# Community perception of the usefulness and ease of COVID-19 self-test in two areas in Paraguay

**DOI:** 10.1590/0037-8682-0381-2024

**Published:** 2025-05-09

**Authors:** Cristina Alonso-Vega, Silvia Pérez, Elizabeth de Jesús Posada, Beatriz Mallén, Jazmín Cabañas, Luis Rodrigo Villarroel, María Victoria Alé, Luis Rolando Vera, Verónica Burgos, Sandra Irala, Nelly Regina Rabinovich

**Affiliations:** 1 ISGlobal, Barcelona, Spain.; 2 ECO Team Paraguay, Asunción, Paraguay.; 3 QUATRIM Sociedad de Responsabilidad Limitada, Cochabamba, Bolivia.; 4 Ministerio de Salud Pública y Bienestar Social, Asunción, Paraguay.

**Keywords:** COVID-19, self-testing, feasibility, community

## Abstract

**Background:**

The coronavirus disease 2019 (COVID-19) pandemic posed challenges for health systems in rapidly diagnosing diseases.

**Methods:**

Community workshops and a self-administered questionnaire were implemented to determine the feasibility of self-testing for future epidemics and gain an understanding of community interest and the capacity to implement self-tests in two rural areas in Paraguay.

**Results:**

Comprehension of the self-test procedures and their application was demonstrated by 99% of the participants. The participants expressed interest in accessing these tests at home.

**Conclusions:**

Self-tests could be a valuable tool in urban and rural communities that enable health systems to focus on severe cases.

The coronavirus disease 2019 (COVID-19) outbreak was declared a pandemic on March 11, 2020, by the World Health Organization (WHO). Waves of infections with varying numbers of cases, severity, and lethality were reported globally. COVID-19 was declared an established and ongoing health issue that no longer constituted a public health emergency of international concern (PHEIC) on May 4th, 2023, by the WHO^1^. Diagnostic capacity played a crucial role in public health and clinical management strategies during the pandemic. Rapid and accurate diagnosis facilitates early treatment, particularly in high-risk individuals. Furthermore, it facilitates the adoption of measures to limit the spread of the virus. 

Real-time reverse transcription polymerase chain reaction (RT-PCR), which has a sensitivity and specificity of 96.2% and 98.1% respectively^2^, was the first tool available for diagnosis. RT-PCR remains the gold standard for the diagnosis of COVID-19; however, it requires well-equipped laboratories and personnel. Although RT-PCR is widely available in high-income countries, its availability is limited in low- and middle-income countries (LMICs). Only five public laboratories located in major cities were equipped to perform PCRs in Paraguay, hindering timely diagnosis^3^. Consequently, diagnosis was difficult during the first wave of the COVID-19 pandemic.

Serological kits based on Enzyme-Linked Immunosorbent Assay (ELISA) have been developed to rapidly detect specific antibodies IgM and IgG against SARS-CoV-2. The overall sensitivity and specificity for IgM were 82% and 97%, respectively, whereas those for IgG were 81% and 98%^2^, respectively. ELISA, which does not require a complex laboratory, is widely implemented. However, the serological test could not detect the first stage of infection.

Rapid antigen detection assays (Ag-RDTs) were developed for the early identification and management of cases. The results of Ag-RDTs are available within 15-30 minutes, and the sensitivity and specificity are 80% and 97%, respectively^4^. 

The first interim guidelines on the role of Ag-RDTs in the diagnosis of COVID-19 in places with limited availability of RT-PCR were released by the WHO on September 11th, 2020. The WHO updated these guidelines on October 6th, 2021, and recommended the use of Ag-RDTs for timely diagnosis^5^, thereby positioning Ag-RDTs as the main tool for identifying active COVID-19 infections. The US Food and Drug Administration (FDA) and the European Center for Disease Prevention and Control (ECDC) recognize self-testing as a powerful tool for controlling the transmission of disease. However, the positive and negative predictive values of self-tests depend on the prevalence of infection, yielding the best results if the incidence is >10%. Another limitation is the difficulty in reporting the results to health authorities. Online systems have been developed to report self-test results and obtain more epidemiological data in some countries^6^. 

The use of COVID-19 self-testing varies across different countries in Latin America, with its use being restricted to an indicative diagnostic tool. However, a positive self-test result was considered a case of COVID-19 if reported to the National System of Health Surveillance in Argentina^7^. Notably, self-testing and self-reporting were approved by the Ministry of Health in Paraguay on January 7th, 2022. However, the Paraguayan Ministry of Health promptly prohibited the use of self-tests owing to a national law relating to the reliance on biochemists to validate the test.

The perception of ease and willingness to use the self-test in two regions in Paraguay was assessed in this study, given the concern voiced by some health authorities over the interest and capacity of the population to implement self-testing. The present study aimed to (i) assess the ability of the population to perform the self-test correctly after a training session, (ii) verify the responses of the participants to the test (positive/negative), (iii) evaluate the interest of the participants in accessing the test at home, and (iv) assessing the feasibility and convenience of taking the tests at home.

This study was conducted at healthcare centers in Presidente Hayes and Cordillera departments in Paraguay, wherein the ISGlobal-ECO project was implemented^8^. Both sites catered to a mixed population of urban and rural individuals. The sample size was calculated using Cochran’s sample size with a confidence level of 95%, a margin of error of 5%, and a variability of 50%, considering the population of each action area. Based on the population parameters, 760 participants were required. The participants were equally divided between the two departments.

Individuals aged ≥18 years belonging to the catchment area of a local public clinic who were willing to provide written informed consent were eligible for inclusion.

Informative sessions on the use of self-test were organized in coordination with the health personnel of the “Family Health Units” (Unidades de Salud de la Familia - USF). Special attention was paid to groups at high risk of developing COVID-19, such as patients with diabetes, patients with hypertension, and pregnant women. These sessions were conducted in Spanish and Guaraní/Yopará to ensure that all participants understood the content. An overview of the self-test, i.e., functionality, appropriate usage, a practical demonstration for conducting the self-test, and how to interpret the results using explanatory cards and supportive materials, as well as videos provided by the self-test company, was provided.

Only 20 individuals were included in each session to promote discussion and answer any questions that could arise. The participants completed an anonymous questionnaire that aimed to evaluate their understanding of the self-test and identify possible barriers to its implementation. The questionnaire comprised questions regarding (i) their understanding of the information given during the session, (ii) their interest in using the test at home, (iii) their understanding of possible actions to be taken if the result was positive, and (iv) other topics that the participant raised concerns about. The participants were offered a maximum of four tests (Flowflex COVID-19 Antigen Home Test, ACON Laboratories, Inc.) to take home after each session, with the recommendation that they should visit the nearest health center if the result was positive.

Data were collected using a paper questionnaire and digitized using the SPSS database. Quantitative data were analyzed using frequency tables, whereas qualitative data were analyzed using the API of CHAT GPT available in the *software* Scispace Copilot. Furthermore, algorithms and Natural Language Processing (NLP) algorithms were acquired from the NLTK library of Python. 

The study protocol was approved by the WHO Ethics Committee (Protocol ID: CERC.0191) and the Paraguayan MoH Ethics Committee (Protocol code: CEI-LCSP Nº 267/070223).

Between May 29, 2023, and June 8, 2023, 775 individuals, comprising 382 (49.3%) from the 19 health facilities in Cordillera and 393 (50.7%) from the seven facilities in Presidente Hayes, participated in the informative session. All participants completed the questionnaire, achieving diversification according to their sex, age, and risk factors (Table 1).

**TABLE 1:** Attendance and Demographics Summary.

Category	Subcategory	Count (n)	Percentage (%)
**Total Attendance**		775	100%
**Origin**	Cordillera (19 Facilities)	382	49%
	Presidente Hayes (7 facilities)	393	51%
**Age Group**	18-25 years	146	19%
	26-45 tears	424	55%
	46-60 years	151	20%
	Over 60 years	58	8%
**Gender**	Female	591	76.3%
	Male	182	23.4%
	Preferred not to answer	2	0.3%
**Risk Factors**	Family member with risk factors	363	43%
	Participant with risk factors	220	26%
	No risk factors	256	30%
	Preferred not to answer	5	1%

Over 99% of the respondents confirmed that they understood what self-tests were, how to use them, and when to use them. Similarly, 99% of the participants expressed a preference for accessing test kits at home, with the majority (87.4%) electing to take four kits with them. Notably, 64% of the respondents identified the presence of symptoms such as fever and headaches as scenarios warranting the use of self-tests, whereas 21.6% reported using the test after contact with a positive case. 

 The presence of symptoms such as fever and headaches and the event of contact with a positive case were identified as circumstances wherein self-tests could be used. These were the four most common responses identified through the qualitative data analysis conducted using the CHAT API (Figure 1).

**FIGURE 1:**
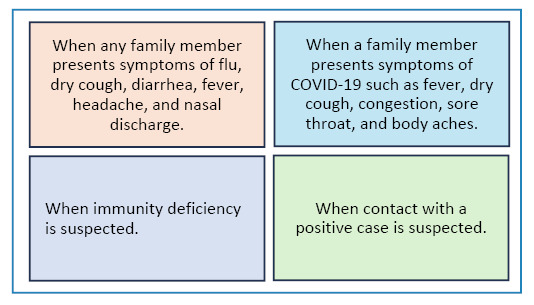
Willingness to use the self-test.

The population exhibited positive behaviors when the results were positive, with four prominent behaviors emerging from the qualitative analysis (Figure 2).

**FIGURE 2:**
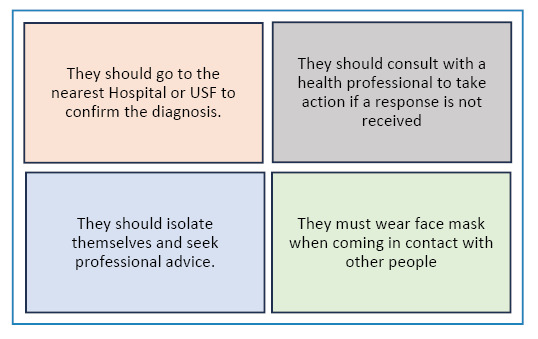
Actions to be taken if the COVID-19 self-test result is positive.

Following the information sessions on the use of self-diagnosis, nearly all participants expressed an interest in using the test at home. They also demonstrated a high level of awareness regarding its usage and an understanding of and willingness to adhere to infection control measures. Notably, 99% of the participants expressed their willingness to perform the COVID-19 self-test when needed at home, which is higher than that reported in other studies. Notably, this percentage varied in Asia: 89.3% in Malaysia, 87.3% in China, 70.3% in the United Arab Emirates, and 60.8% in Indonesia. The highest percentage in Africa was observed in South Africa (90.4%), followed by Nigeria (84.1%) and Kenya (81.4%)^9^. The only data available in the Americas were from Brazil, with a strikingly low 49% of participants expressing willingness to use the self-test^10^. Individuals with secondary education or higher and those with full-time employment in Brazil are more likely to use the self-test. However, the findings of the present study could not be compared with those from Brazil as the employment status and level of education were not recorded in the present study. Nevertheless, the high percentage observed herein may be attributed to the participants engaging in the informative sessions before the survey, which provided them with the opportunity to address concerns regarding the self-test. This close interaction may have contributed to a better understanding of when to use the self-test and how to respond to a positive result. 

Self-diagnosis is broadly applied in a health context, from pregnancy tests that appeared in 1976, through pulse oximeters widely used during the COVID-19 pandemic, to HIV self-tests approved in 2012^11^. Furthermore, self-diagnostic devices have been used to detect noncommunicable diseases, such as glucometers for diabetes and tensiometers for hypertension (HTA). 

Self-testing during a pandemic represents an excellent tool for managing cases at the individual and health system levels. Self-tests enable individuals to make decisions regarding their health by isolating themselves, adopting protection barriers, or seeking medical treatment, as appropriate. These actions reduce the strain on healthcare facilities by enabling the management of mild cases at home, thereby allowing healthcare facilities to focus on more severe cases and continue caring for individuals with chronic diseases.

This is the first study on the use of self-testing conducted in Paraguay. Furthermore, it is one of the few reports available in the Latin American region. This study conducted in rural and urban areas of Paraguay, focusing on individuals with access to public health facilities, revealed a willingness to use COVID-19 self-tests. However, these results may not be fully representative of the entire country as important parts of the population were not represented. In addition, socioeconomic data or the willingness to pay for the self-tests was not captured in the present study. The feasibility of purchasing the tests is uncertain, given the low incomes in the areas where the study was conducted. 

The present study demonstrated that self-tests are acceptable and can be used in both urban and rural communities during pandemics, thereby enabling health systems to focus on severe cases. Through the involvement of the community in the process and boosting of active communication, the use of self-tests can empower the population to manage their health and alleviate the burden on diagnostic health systems. The findings of this study were provided to the Ministry of Health in Paraguay to facilitate a better understanding of the role of self-testing. However, the self-test results can only be considered as indicative results according to the Paraguayan drug regulatory agency (DINAVISA- Resolución 307/2021). 

Self-test-diagnosed cases may not be reported to the health system. Moreover, individuals who test positive may not seek care.

This decentralized approach can accelerate the identification of potential outbreaks and reduce the strain on centralized healthcare facilities, thereby enabling efficient resource allocation. 

## ETHICAL APPROVAL

The ECO Project was approved by the Ethics Committee of CEADES (CE-ECO-17032023, Bolivia) and Hospital Clínic Barcelona (CEIm: HCB/2023/0139, Spain). The participants in the data collection activities provided written informed consent. Confidentiality was assured and all data were managed according to data protection regimens (including General Data Protection Regulation [GDPR]).
